# A Preliminary Environmental Assessment of Epoxidized Sucrose Soyate (ESS)-Based Biocomposite

**DOI:** 10.3390/molecules25122797

**Published:** 2020-06-17

**Authors:** Shokoofeh Ghasemi, Mukund P. Sibi, Chad A. Ulven, Dean C. Webster, Ghasideh Pourhashem

**Affiliations:** 1 Department of Coatings and Polymeric Materials, North Dakota State University, Fargo, ND 58108, USA; Shokoofeh.Ghasemi@ndsu.edu (S.G.); Dean.Webster@ndsu.edu (D.C.W.); 2 Department of Chemistry and Biochemistry, North Dakota State University, Fargo, ND 58108, USA; Mukund.Sibi@ndsu.edu; 3 Department of Mechanical Engineering, North Dakota State University, Fargo, ND 58108, USA; Chad.Ulven@ndsu.edu

**Keywords:** epoxidized sucrose soyate (ESS), life cycle assessment, biocomposite, soybean-based resin

## Abstract

Biocomposites can be both environmentally and economically beneficial: during their life cycle they generally use and generate less petroleum-based carbon, and when produced from the byproduct of another industry or recycled back to the manufacturing process, they will bring additional economic benefits through contributing to a circular economy. Here we investigate and compare the environmental performance of a biocomposite composed of a soybean oil-based resin (epoxidized sucrose soyate) and flax-based reinforcement using life cycle assessment (LCA) methodology. We evaluate the main environmental impacts that are generated during the production of the bio-based resin used in the biocomposite, as well as the biocomposite itself. We compare the life cycle impacts of the proposed biocomposite to a functionally similar petroleum-based resin and flax fiber reinforced composite, to identify tradeoffs between the environmental performance of the two products. We demonstrate that the bio-based resin (epoxidized sucrose soyate) compared to a conventional (bisphenol A-based) resin shows lower negative environmental impacts in most studied categories. When comparing the biocomposite to the fossil fuel derived composite, it is demonstrated that using epoxidized sucrose soyate versus a bisphenol A (BPA)-based epoxy resin can improve the environmental performance of the composite in most categories except eutrophication and ozone layer depletion. For future designs, considering an alternative cross-linker to facilitate the bond between the bio-based resin and the flax fiber, may help improve the overall environmental performance of the biocomposite. An uncertainty analysis was also performed to evaluate the effect of variation in LCA model inputs on the environmental results for both the biocomposite and composite. The findings show a better overall carbon footprint for the biocomposite compared to the BPA-based composite at almost all times, demonstrating a good potential for marketability especially in the presence of incentives or regulations that address reducing the carbon intensity of products. This analysis allowed us to pinpoint hotspots in the biocomposite’s supply chain and recommend future modifications to improve the product’s sustainability.

## 1. Introduction

Conventional polymeric resins are often composed of several hazardous materials such as isopentane and isoprene, i.e., in aliphatic petroleum resin, or benzene, i.e., in aromatic types [1]. Additionally, increasing environmental concerns due to production and use of conventional petroleum-based products, in the past few decades, have sparked a growing interest for renewable, less hazardous and sustainable resins. Extensive research has been conducted on the production of bio-based resins that can meet sustainability requirements while providing similar or enhanced functionalities compared to petroleum-based resins [2]. Epoxidized vegetable oils and their derivatives have been recognized as potentially sustainable alternative sources with comparable properties to petroleum-based resins [3]. Among epoxidized vegetable oil-based resins, epoxidized sucrose soyate (ESS) has shown great potential as it and its derivatives have been shown to significantly enhance the mechanical properties of thermosets in a wide range of applications including composites [4,5,6]. A study of preparing a biocomposite from highly functional methacrylated epoxidized sucrose soyate and fiberglass fabric by Hosseini et al. (2016) demonstrated that the produced biocomposite had excellent interfacial and mechanical properties [7]. ESS has also been used to overcome the poor properties of vegetable oil-based resins in composites by exhibiting lower degradation in mechanical properties and appearance when the composite was exposed to accelerated weathering [8].

Though inherently bio-based, it is essential to ensure these resins and the biocomposites can offer more sustainable alternatives for products they intend to replace. A few studies have investigated the environmental performance of bio-based resins and composites [9,10,11,12,13], where those resins and composites demonstrated favorable environmental impacts including carbon footprint and energy use compared to their petroleum-based counterparts. However, despite the growing research and interest in the resins and composites made from renewable materials, studies on the sustainability of these materials are limited. The goal of this study is to investigate whether ESS, while providing equivalent functionality to conventional resins used in composites, can mitigate their associated environmental impacts. To the best of our knowledge, this is the first study to investigate the environmental impacts of ESS.

To ensure that ESS and the ESS-based biocomposite do not generate unintended environmental or health impacts during their production, a comprehensive assessment of the sustainability of ESS thermosetting resin and their biocomposites throughout their life cycle is needed. Life cycle assessment (LCA) is a holistic assessment tool that is used in this study to evaluate how the ESS-based biocomposite potentially affects ecosystems over its entire life cycle starting from the extraction of the raw materials to the end-of life stage of the biocomposite manufacturing. Life cycle assessment is a method of examining all contributors to the overall environmental impact of a product. LCA can be used as a guide when designing or refining the synthesis of a material, helping in selecting materials and process conditions that can result in optimal environmental performance. Here, we build an LCA model for the production of the ESS thermosetting resin and the biocomposites made with ESS as an alternative to bisphenol A (BPA)-based resins, and compare their environmental performance with functionally similar conventional materials in the market. The main goal of this study is to carry out a preliminary LCA study that will allow us to identify hotspots in the production process and recommend possible improvements and waste minimization during its production that can help this novel thermosetting resin compete with commercially available resins in the market.

## 2. Materials and Methods

We applied LCA following International Organization for Standardization (ISO) methods [14] to investigate the environmental impacts of ESS-based biocomposite. Our LCA model consists of two main stages: making (1) ESS, and (2) ESS-based composites. We built the ESS model based on the primary data from a 10 kg pilot-scale production of ESS [15]. For the biocomposite production stage, we examined a case of ESS used in a biocomposite where it is reinforced with flax fiber to functionally replace commercially available BPA-based resins [8]. SimaPro software [16] was used to build the LCA model, and to evaluate and compare the environmental performance of the studied bio-based composite with a petroleum-based composite.

### 2.1. Goal and Scope of the Study

The goal of this study is to evaluate and compare the life cycle environmental impacts of ESS resin and flax-based reinforced biocomposite with similar performance to conventional BPA-based resins and flax fiber reinforced composite. The biocomposite we used for our LCA model was prepared by using ESS, flax fiber, cross-linker and catalyst. The petroleum-based composite was prepared using BPA epoxy resin with hardener and flax fiber. The amounts of flax fiber used per kg of final product is similar for both the studied biocomposite and the petroleum-based composite. The models for the final manufacturing step of both composites were built based on formulations in a study by Taylor et al. (2016) [8]. We selected two formulations that were representative of all formulations tested in that study that exceeded their desired mechanical properties.

**Figure 1:**
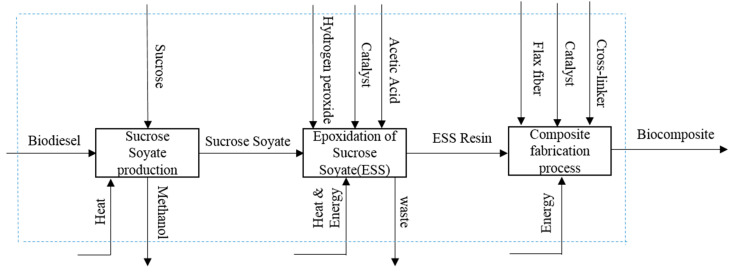
Process flow for the production of ESS and ESS-based biocomposite, the dashed line indicates the system boundary.

ESS resin is produced via epoxidation of sucrose soyate, which is produced from transesterification of methyl ester of soybean oil and sucrose. The system boundary for this LCA study includes: (1) production of ESS resin, and (2) production of ESS-based biocomposite (Figure 1). Production of ESS resin in our model consists of (1) transesterification of methyl ester of soybean oil (Biodiesel) and sucrose to generate sucrose soyate, and (2) epoxidation of sucrose soyate. Soybean is a dominant oil seed in the U.S. and in the past three years, the U.S. has been the largest producer of soybean oil in the world [17]. Therefore, the U.S. is considered the geographical boundary of the life cycle. The selected functional unit for this study is 1 kg of composite.

### 2.2. Life Cycle Inventory and Impact Assessment

**Figure 2:**
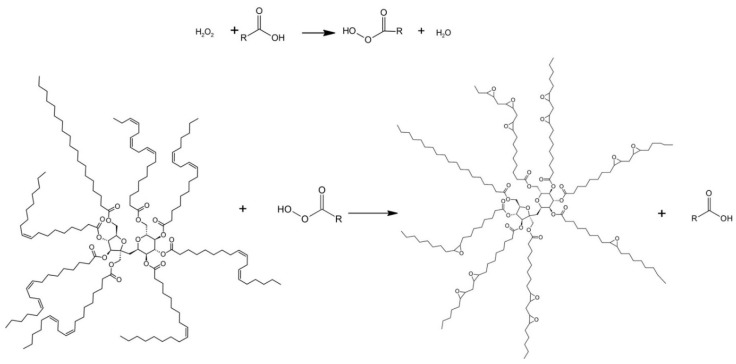
Epoxidation process of sucrose soyate [15].

Sucrose soyate is epoxidized to ESS using peracetic acid formed from the reaction of acetic acid and hydrogen peroxide. A schematic representation of this process is displayed in Figure 2 [15]. Epoxidation of sucrose soyate proceeds with more than 98% conversion yielding ESS. Data for the construction of the life cycle inventory (LCI) model include the material and energy inputs to (1) produce sucrose soyate, (2) epoxidize sucrose soyate, and (3) crosslink ESS with flax fiber to produce the biocomposite. The inputs to sucrose soyate production were taken from Granberg and Schafermeyer [18] and the inputs for modeling the epoxidation of sucrose soyate (Table 1) were based on a 10 kg pilot study by Monono et al. [15]. The information for the production of methyl ester of soybean oil was taken from the Ecoinvent v.3 database [19]. Data for flax fiber production and biocomposite manufacturing were taken from Dissanayake et al. [20] and Taylor et al. [8], respectively. Full details of the inputs to the life cycle model are provided in Table S1 [see Supporting Information]. In our model, we assume that the catalyst (Amberlite) is recycled and returned to the process. In all of the life cycle steps, the exact amount of some components and energy consumption were not specified, therefore we calculated the missing information based on the reported data [15,18]. During the sucrose soyate production step, methanol is generated as a byproduct, which is considered to generate a credit for the system. The produced resin is used in a biocomposite with flax fiber to replace BPA-based resins, where it provides comparable properties [8].

The Tool for reduction and assessment of chemical and other environmental impacts (TRACI) 2 method (including ten environmental impact categories, e.g., global warming) was selected to investigate the environmental performance of our system. Additionally, we accounted for the biogenic carbon content of sucrose soyate as the carbon was sequestered from the atmosphere and was therefore a credit for our studied system. The amount of carbon credit was calculated based on the amount of carbon content of sucrose soyate, which equals 2.75 g CO_2_/g of sucrose soyate.

## 3. Results and Discussion

### 3.1. Environmental Impact of ESS Resin and ESS-Based Biocomposite

**Figure 3:**
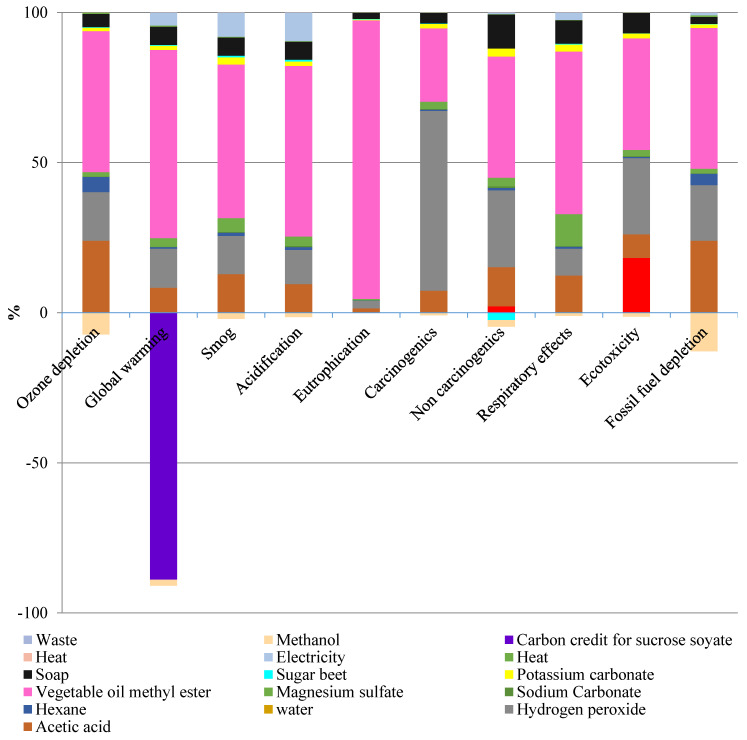
Life cycle environmental impacts of producing 1 kg of ESS using TRACI 2 method.

A simplified profile of contributions to environmental impacts of ESS production is depicted in Figure 3. Ten impact categories studied in this assessment were ozone depletion, global warming, smog, acidification, eutrophication, carcinogenics, non-carcinogenics, respiratory effects, eco-toxicity, and fossil fuel depletion. In most categories, vegetable oil methyl ester used for producing sucrose soyate had the largest contribution to the impacts as one of the main inputs for this product. Next, acetic acid and hydrogen peroxide had the most impact among reagents while hydrogen peroxide was the reagent with most carcinogenic impact, with its figure standing at 60% of this category.

Hexane and anhydrous magnesium sulfate had the least impact overall, accounting for less than 15% of impacts in all categories. The highest contribution of anhydrous magnesium sulfate was to respiratory effects, which can be eliminated in an industrial scale production. In the pilot-scale study, magnesium sulfate was used as a drying agent, whereas the drying method will be replaced by industrial sized driers in a large-scale production.

As can be seen from Figure 3, biogenic CO_2_ credit for sucrose soyate accounted for more than 90% of the impact in this category, which reduced the global warming impact significantly. In bio-based feedstocks, atmospheric carbon is first absorbed into the molecular chains of biomass in cultivation, stored during bio-based product use, and then released into the air via decomposition, or sequestered in the product at the end of life depending on the product application [10]. Contribution of all inputs to greenhouse gas (GHG) emissions are presented in Table S2. Methanol produced as a byproduct in the sucrose soyate production stage also generated a credit in some categories, such as ozone layer depletion and fossil fuel depletion.

**Figure 4:**
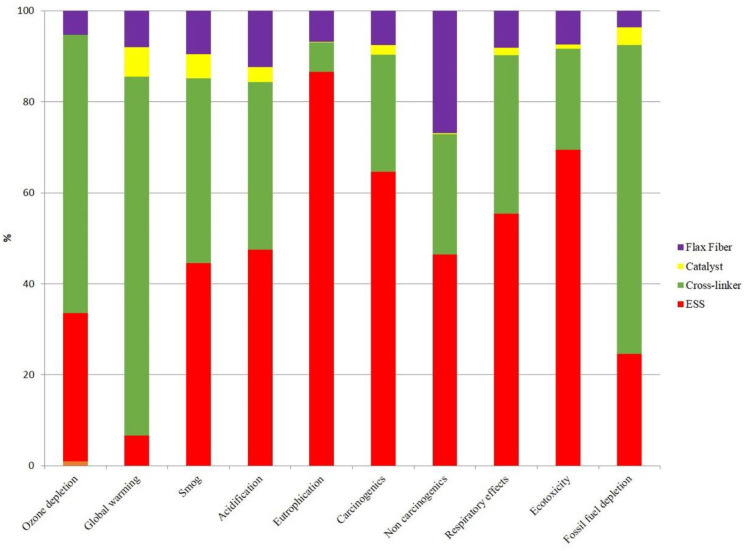
Contribution of biocomposite’s constituents to examined environmental impacts.

Similarly, we ran a full LCA model for the ESS-based biocomposite production (Figure 4). Results showed that the ESS production step accounts for most environmental impacts in all categories except ozone depletion, global warming and fossil fuel depletion. The cross-linker has the main contribution in these three categories, equal to about 61, 79 and 68%, respectively, over the full life cycle of the biocomposite.

### 3.2. Comparison of Bio-Based and Conventional Resins and Composites

**Figure 5:**
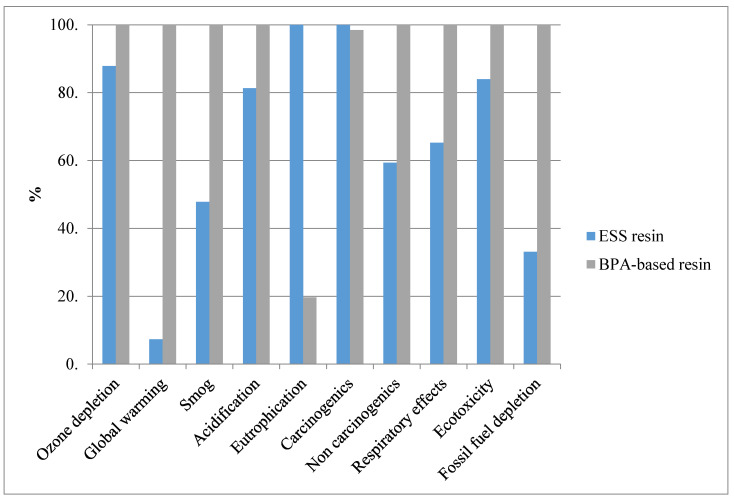
Comparing 1 kg ESS resin with 1 kg BPA-based resin.

Figure 5 shows a comparison between the environmental impacts of producing 1 kg of ESS resin and 1 kg of a BPA-based epoxy resin (Araldite LY 8601). A significantly lower impact in all categories except eutrophication and carcinogenics can be seen for ESS resin versus BPA-based resin. The high impact of ESS in the eutrophication category could be attributed to the emissions from pesticides and fertilizers used during soybean cultivation. Better farm management practices during soybean production, such as efficient use of fertilizers, can help improve the upstream life cycle impacts of the soy-based products. Although, in this preliminary study methyl ester of soybean oil was considered as a main input in the model from the Ecoinvent database, to find the accurate sources of emissions of this input, a cradle-to-gate boundary that models the production of soybean and its processing to methyl ester in detail should be analyzed. The high impact in the carcinogenics category of ESS comes from the use of hydrogen peroxide as discussed earlier. The impact associated with the fossil fuel depletion by ESS was 33%, which is one-third of the fossil fuel used when BPA-based resin is produced. BPA-based resin’s contribution to global warming was 3.92 kg CO_2_ eq/kg of Araldite 8601, whereas, the figure for ESS stood at 0.287 kg CO_2_ eq/kg of ESS. This translates to a substantial reduction in global warming by bio-based resin. The impact of ESS production on global warming is significantly lower compared to other bio-based thermosetting resins reported elsewhere. For example, Chard et al. (2019) conducted research on a thermoset resin in which some of the petrochemical-based raw materials were substituted with vegetable oils providing similar mechanical performance compared to the standard thermosetting resin system. Their study showed a life cycle greenhouse gas emission of 5.7 kg CO_2_ per kg of resin [21]. In another study, Rosa et al. (2014) reported 4.079 kg CO_2_ eq per kg of their bio-based epoxy resin (SuperSap 100/1000) in global warming potential [22].

**Figure 6:**
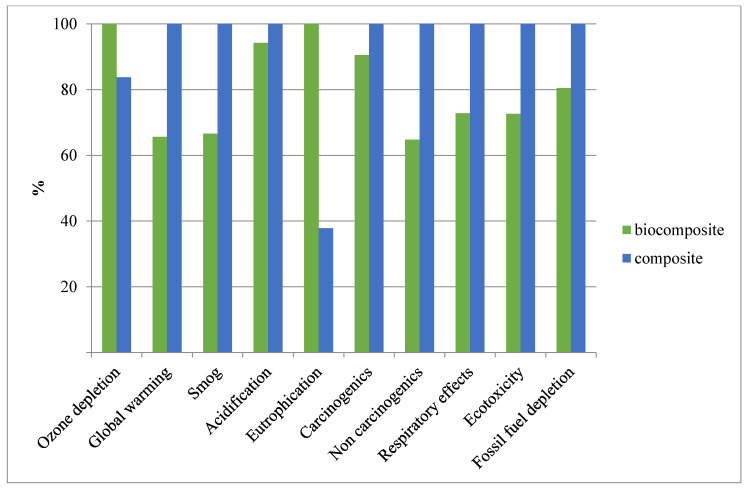
Comparing the environmental impacts of producing 1 kg of biocomposite with 1 kg of BPA-based composite using the TRAI 2 method.

In the next step, we conducted an environmental impact comparison analysis for producing 1 kg of biocomposite made with ESS and 1 kg of a conventional (BPA-based) composite (Figure 6). The resin (Araldite LY 8601) and hardener (Aradur 8602) in composite components were built in the LCA model based on Huntsman’s 2010 product factsheet [23]. Since the exact details of the production process is kept confidential and not published, we used the average amounts of components for building the model. As can be observed from Figure 6, the biocomposite illustrated better environmental impacts in most categories relative to the conventional composite. However, in the ozone depletion category, the biocomposite’s impact surpassed the conventional composite’s impact. The additional 17% higher impact of biocomposite in the ozone depletion category is due to the use of the cross-linker (methyl hexahydrophthalic anhydride) for the preparation of the biocomposite. Changing the cross-linker to one that is functionally similar but with lower environmental impacts can reduce the contribution of this component and improve the life cycle performance of the biocomposite. The inventory for building the LCA model of the cross-linker is provided in Table S3 based on [24,25]. As can be observed, the biocomposite also demonstrated no impact reduction in the eutrophication category. The high impact in this category originates from the production of ESS, as discussed earlier. However, the impact in other categories, such as global warming, smog, non-carcinogenics, and respiratory effects eco-toxicity, was diminished by replacing BPA-based resin in the composite. The conventional composite’s contribution to global warming was 1.78 kg CO_2_ eq/kg of composite, whereas, the biocomposite’s global warming potential was lower, showing 1.17 kg CO_2_ eq/kg of biocomposite (over 30% lower). Thus, the decrease in contribution of biocomposite to global warming can be attributed to the biogenic CO_2_. In the presence of incentives or regulations that address reducing the carbon footprint of products, ESS-based biocomposite can offer a better alternative to the conventional BPA-based composites. The absolute values of contribution for all categories are available in Table S4.

### 3.3. Uncertainty in LCA Outcomes

**Figure 7:**
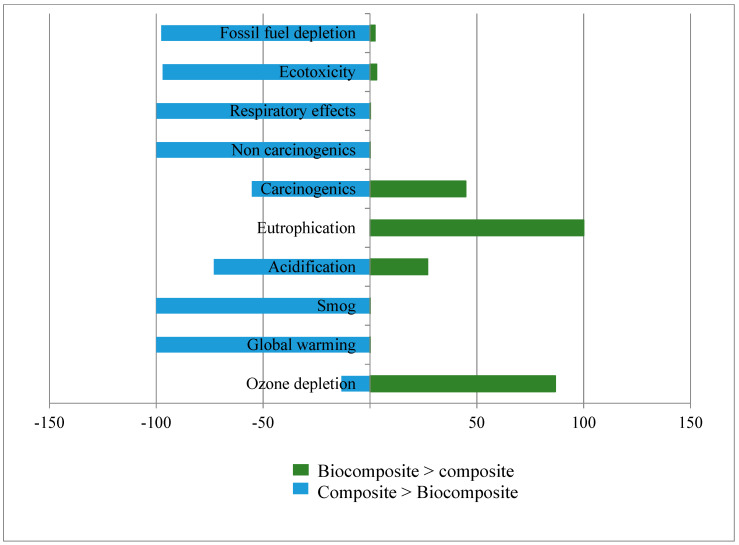
Uncertainty results for comparing biocomposite and petroleum-based composite, with a 95% confidence interval, demonstrating the likelihood of composite or biocomposite having higher or lower impact in each category.

To assess the robustness and transparency of LCA outputs, and to provide better decision-making information, we conducted uncertainty analysis using SimaPro software [16]. The Monte Carlo technique was applied with 10,000 iterations at the 95% confidence level to compare the uncertainty in the biocomposite and petroleum-based composite LCA results (Figure 7). The results display a higher probability for biocomposite outperforming petroleum-based composite in most impact categories. However, biocomposite revealed higher impact in eutrophication and ozone depletion categories with 87.67% and 99.99% probability, respectively. The higher probability of biocomposite exhibiting higher impact in these two categories than composite is compatible with our deterministic results from Section 3.2. High confidence in LCA results can therefore be concluded by this evaluation.

**Figure 8:**
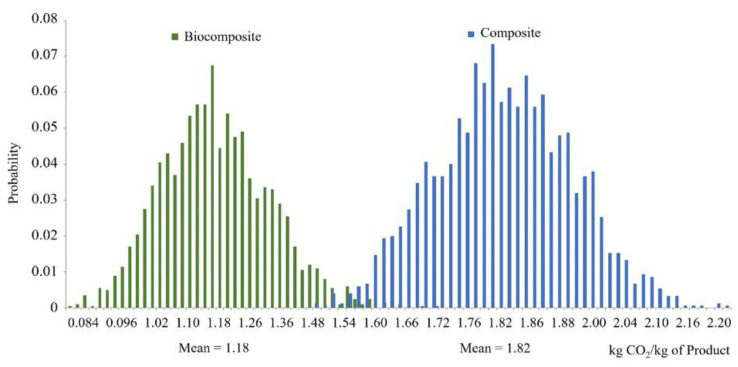
Probability distribution of Monte Carlo simulation results for global warming potential of biocomposite and petroleum-based composite, with 95% confidence interval.

Carbon footprint is the most well-known and common metric to evaluate the performance of bio-based materials in contrast to fossil-based materials. Therefore, we further analyzed the two samples (petroleum-based composite and biocomposite total greenhouse gas (GHG) emissions) to ensure they are statistically different. Figure 8 shows the results of LCA uncertainty model runs for the global warming potential of biocomposite and petroleum-based composite as a probability distribution function. The results of a t-test we performed on the two GHG emission samples demonstrated a *p* < 0.001, which means there is a significant difference in GHG emissions between the two composites. Thus, petroleum-based composite always has a higher global warming potential (GWP) impact than biocomposite (over 50% higher).

## 4. Conclusions

To study the sustainability of ESS resin and ESS-based biocomposite, we built and conducted an LCA study of these products in comparison with BPA-based composites. We created a model with a cradle-to-gate system boundary using SimaPro software to identify the hotspots in the production of the ESS-based biocomposite. The results showed that, for ESS production, the greatest contribution in almost all environmental impact categories was due to the vegetable oil methyl ester. Moreover, hydrogen peroxide indicated a high impact in the carcinogenic category.

Additionally, compared to petroleum-based (BPA-based) resin, the bio-based resin demonstrated an overall reduction of environmental footprint except in the eutrophication and carcinogenics impact categories, mostly due to vegetable oil methyl ester production and the use of hydrogen peroxide. This is a preliminary study and the exact source of emissions for farm-level activities are not assessed in this model. Therefore, a future more detailed cradle-to-gate or cradle-to-grave study is necessary to provide additional information on the upstream and end-of-life emissions.

A comparison between a composite derived from bio-based resin (ESS) and a petroleum-derived resin (BPA-based) revealed that a biocomposite can potentially mitigate many of the environmental impacts associated with a conventional composite. The cross-linker, methyl hexahydrophthalic anhydride, used in the studied biocomposite showed a negative impact in the ozone depletion category. Further investigation is needed to find an alternative for alleviating the cross-linker’s negative impact in this category.

We used Monte Carlo analysis to test the uncertainty in the LCA results for all impact categories. A comparison between the GWP of the two composites showed that they are significantly different from the petroleum-based composite having a higher carbon footprint compared to the biocomposite at almost all times. To conclude, ESS resin can provide a viable alternative to conventional resins while reducing its overall environmental footprint in most impact categories. In addition, the ESS resin can be considered as an alternative to BPA-based resins in making biocomposites, particularly if a substitute cross-linker with lower contribution to ozone layer depletion is used.

## Figures and Tables

**Table 1:** LCI data for 10 kg of ESS production.

	Inputs		Output	
	Unit	Quantity	Unit	Quantity
ESS ^1^			kg	9.825
Sucrose soyate ^2^	kg	10		
Acetic Acid	g	1575	g	1230
Amberlite IR120 H ^1^	g	2000	g	2000
Hydrogen peroxide ^1^	g	3410	g	1500
Water ^2^	g	60,000		
Hexane ^2^	g	2620	g	1703
Sodium carbonate ^2^	g	250	g	0
Magnesium sulfate ^1^	g	2000	g	2000
Sodium acetate ^3^			g	287
Electricity	J	1,062,120		
Heat	J	54,286		

^1^ Calculated based on Monono et al., 2015 [15]; ^2^ Monono et al., 2015 [15], ^3^ Salt generated when remaining acetic acid in the resin is neutralized with sodium carbonate.

## Supplementary Materials

The following are available online. Table S1: LCI for the whole process of ESS production, Table S2: Impact assessment on Global warming of ESS, Table S3: LCI for Cross-linker, Table S4: Absolute values for composite and biocomposite impact in different environmental categories.
