# Supplementary material for: A Preliminary Environmental Assessment of Epoxidized Sucrose Soyate (ESS)-Based Biocomposite
Source: Molecules. 2020 Jun 17;25(12):2797. doi: 10.3390/molecules25122797 (PMC7356212; doi:10.3390/molecules25122797)
Supplement: Supplementary file 1 [file molecules-25-02797-s001.pdf]

Supplmentary Materials

A Preliminary Environmental Assessment of Epoxidized Sucrose Soyate (ESS)-Based Biocomposite

Shokoofeh Ghasemi [1], Mukund P. Sibi [2], Chad A. Ulven [3], Dean C. Webster [1] and Ghasideh Pourhashem [1,*]

[1] Department of Coatings and Polymeric Materials, North Dakota State University, Fargo, ND 58108, USA; Shokoofeh.Ghasemi@ndsu.edu (S.G.); Dean.Webster@ndsu.edu (D.C.W.)

[2] Department of Chemistry and Biochemistry, North Dakota State University, Fargo, ND 58108, USA; Mukund.Sibi@ndsu.edu

[3] Department of Mechanical Engineering, North Dakota State University, Fargo, ND 58108, USA; Chad.Ulven@ndsu.edu

* Correspondence: ghasideh.pourhashem@ndsu.edu

Supporting information:

Table 1. LCI for the whole process of ESS production.

	Inputs		Output	
	Unit	Quantity	Unit	Quantity
ESS[1]			kg	9.825
Sucrose soyate[2]	kg	10		
Acetic Acid		1575	g	1230
Amberlite IR120 H[1]	g	2000	g	2000
Hydrogen peroxide[1]	g	3410	g	1500
Water[2]	g	60000		
Hexane[2]	g	2620	g	1703
Sodium carbonate[2]	g	250		0
Magnesium sulfate[1]	g	2000	g	2000
Sodium acetate[3]			g	287
Electricity	kJ	7.3854		
Heat for epoxidizing[2]	kJ	26.553		
Methanol[4]			g	1012.5
Vegetable oil methyl ester[4]	g	12750		
Potassium carbonate[4]	g	150		
Sugar from sugar beat[4]	g	1350		
Soap[3]	g	750		
Heat for sucrose soyate production[4]	kJ	37.013		

1-Calculated based on Monono et al., 2015[1]; 2-Monono et al., 2015[1]; 3-Calculated based on neutralizing remaining acetic acid; 4-Calculated based on Granberg et al,1997 [2].

Table 2. Impact assessment on Global warming of ESS.

Inputs	kg CO_2 eq/ kg of ESS
Acetic Acid	0.263
Hydrogen Peroxide	0.409
Water	8.05E-05
Hexane	0.0162
Soda Ash	0.00417
Magnesium Sulfate	0.0891
Vegetable Oil Methyl Ester	1.97
Potassium Carbonate	0.0446
Sugar Beet	0.00738
Soap	0.193
Heat	0.011
Electricity	0.136
Heat	0.000384
Carbon Credit for Sucrose Soyate	-2.8
Methanol	-0.0628
Total	0.287

Table 3. LCI for Cross-linker [3].

	Inputs (g)	Outputs (g)	Reference
methyl hexahydrophthalic anhydride		152	[3]
Aluminum chloride	3		Ecoinvent v.3
Maleic anhydride	98		Ecoinvent v.3
isoprene	47.26		[4]

Table 4. Absolute values for composite and biocomposite impact in different environmental categories.

Impact category	Unit	Composite	Biocomposite
Ozone depletion	Kg CFC -11 eq	2.14E-7	2.56E-7
Global warming	Kg CO2 eq	1.78	1.17
Smog	Kg O3 eq	0.107	0.0712
Acidification	Kg SO2 eq	0.00792	0.00746
Euthrophication	Kg N eq	0.00771	0.0204
Carcinogenics	CTUh	9.45E-8	8.5E-8
Non-carcinogenics	CTUh	5.55E-7	3.59E-7
Respiratory effects	Kg PM2.5 eq	0.00146	0.00106
Ecotoxicity	CTUe	14.8	10.7
Fossil fuel depletion	MJ surplus	4.8	3.86

References:

1. Monono, E.M.; Webster, D.C.; Wiesenborn, D.P. Pilot scale (10kg) production and characterization of epoxidized sucrose soyate. *Ind. Crops Prod.* **2015**, *74*, 987–997
2. US5648483A-Continuous Transesterification Method for Preparing Polyol Polyesters-Google Patents. Available online: https://patents.google.com/patent/US5648483A/en (accessed on 25 March 2020).
3. Libing, G.; Haiyang, Z.; Dong, Z.; Ruina, Y.; Liang, Z.; Pengfei, F.; Zhenfeng, X. Novel Method of Producing Methyl Hexahydrobenzene Anhydride. Chinese patent CN1313456C, 2 May 2007.
4. Morais, A.R.C.; Dworakowska, S.; Reis, A.; Gouveia, L.; Matos, C.T.; Bogdał, D.; Bogel-ŁUkasik, R. Chemical and biological-based isoprene production: Green metrics. *Catal. Today* **2015**, *239*, 38–43.
